# What Is on the Horizon for Treatments in Idiopathic Pulmonary Fibrosis?

**DOI:** 10.3390/jcm13216304

**Published:** 2024-10-22

**Authors:** Teng Moua, Misbah Baqir, Jay H. Ryu

**Affiliations:** Division of Pulmonary and Critical Care Medicine, Mayo Clinic, 200 First St SW, Rochester, MN 55905, USA; baqir.misbah@mayo.edu (M.B.); ryu.jay@mayo.edu (J.H.R.)

**Keywords:** therapeutics, treatment, idiopathic pulmonary fibrosis, lung fibrosis

## Abstract

Idiopathic pulmonary fibrosis (IPF) is a progressive and often fatal lung disease most commonly encountered in older individuals. Several decades of research have contributed to a better understanding of its pathogenesis, though only two drugs thus far have shown treatment efficacy, i.e., by slowing the decline of lung function. The pathogenesis of IPF remains incompletely understood and involves multiple complex interactions and mechanisms working in tandem or separately to result in unchecked deposition of extracellular matrix components and collagen characteristic of the disease. These mechanisms include aberrant response to injury in the alveolar epithelium, inappropriate communication between epithelial cells and mesenchymal cells, imbalances between oxidative injury and tissue repair, recruitment of inflammatory pathways that induce fibrosis, and cell senescence leading to sustained activation and proliferation of fibroblasts and myofibroblasts. Targeted approaches to each of these mechanistic pathways have led to recent clinical studies evaluating the safety and efficacy of several agents. This review highlights selected concepts in the pathogenesis of IPF as a rationale for understanding current or future therapeutic approaches, followed by a review of several selected agents and their recent or active clinical studies. Current novel therapies include approaches to attenuating or modifying specific cellular or signaling processes in the fibrotic pathway, modifying inflammatory and metabolic derangements, and minimizing inappropriate cell senescence.

## 1. Introduction

Idiopathic pulmonary fibrosis (IPF) is a progressive and often fatal disease characterized by the accumulation of fibrotic tissue in the lung [1]. While two antifibrotic medications (nintedanib and pirfenidone) [2,3] have been shown to slow disease progression and are currently in use, a significant need remains for more effective agents that may prevent loss of lung function and even reverse fibrosis. Adverse effects and long-term adherence remain difficult and may confound real-world effectiveness, despite long-term use demonstrating sustained lung function benefit, decreased exacerbation rate, or better mortality outcomes in certain studies [4,5]. Over the past two and a half decades, multiple clinical trials have assessed the efficacy of various agents, including azathioprine in combination with prednisone (anti-inflammatory) [6], interferon-γ (immune response regulator) [7], etanercept (tumor necrosis factor-receptor (TNF-α) inhibitor) [8], macitentan [9], bosentan [10], and ambrisentan [11] (endothelin receptor antagonists), sildenafil (phosphodiesterase-5 inhibitor) [12], imatinib mesylate (tyrosine kinase inhibitor) [13], N-acetylcysteine (anti-oxidant) [14], warfarin (anti-coagulant) [15], ziritaxestat (autotaxin inhibitor) [16], simtuzumab (lysyl oxidase inhibitor) [17], and pamrevlumab (human monoclonal antibody against connective tissue growth factor (CTGF)) [18], all with little-to-no impact on lung function or other secondary outcomes. Recent research has focused on additional molecular targets in the cell signaling of fibrotic pathways [19,20], oxidative stress and inflammatory responses [21], and cell senescence [22,23], with the potential combination of currently established and novel therapies for synergistic effect. The first section of this review broadly highlights concepts in the pathogenesis of IPF that underlie current therapeutic approaches. The second section provides updates on recently tested medications based on these concepts for a better understanding of current drug rationale and future therapeutic directions.

## 2. Selective Overview of IPF Pathogenesis

IPF is characterized by the inappropriate or unchecked deposition of collagen and extra-cellular matrix (ECM) in lung tissue. This is the result of dysregulated host responses at the alveolar epithelium [24], excessive proliferation and propagation of pro-fibrotic cells either by sustained cytokine or chemokine-driven processes and inappropriate cell senescence [23,25], and imbalances between propagating and degrading fibrotic response [26]. Abnormal cell repair or recovery from oxidative stress or inflammation also contribute to pro-fibrotic processes [27]. An overview of selected pathologic mechanisms is provided in Table 1.

### 2.1. Abnormalities at the Alveolar Epithelium and Alveolar Epithelial Cells

Injury to the alveolar epithelium has long been a point of interest as an initial trigger of parenchymal fibrosis [24,28]. Alveolar epithelial cells (AECs) produce surfactant and play central roles in maintaining alveolar integrity and directing cell repair after injury [29]. Damage to type II AECs leads to the release of pro-fibrotic mediators, particularly transforming growth factor-beta (TGF-β) [30,31,32], responsible for multiple downstream fibrotic effects. Epithelial-mesenchymal transition (EMT), characterized by the transformation of epithelial cells to mesenchymal phenotypes [33], is another mechanism by which AECs contribute to lung fibrosis [34,35,36]. TGF-β signaling is a driver of EMT and leads to fibroblast imitation and activation [37,38]. AECs are also susceptible to oxidative damage from environmental or inflammatory mediators [39,40]. Prior studies have demonstrated oxidative stress may induce cellular senescence in AECs [41], impairing their regenerative capacity and promoting a pro-fibrotic environment [42]. The accumulation of senescent AECs has been associated with mitochondrial dysfunction [43]. Lastly, AECs secrete various cytokines and chemokines that influence the behavior of macrophages [44], promoting their activation and contribution to an inflammatory milieu that promotes fibrotic response.

### 2.2. TGF-β, Fibroblasts, and Myofibroblasts

TGF-β is a multifaceted cytokine involved in various cellular processes in the lung, including EMT, fibroblast activation, and ECM deposition [45]. It is primarily secreted by AECs [30] and activated macrophages [46,47], with its dysregulation leading to unchecked activation of fibroblasts and myofibroblasts. TGF-β also activates various downstream pathways including Smad signaling which mediate transcriptional responses involved in fibroblast proliferation and differentiation [48,49]. Interaction between TGF-β and other fibroblast growth factor pathways has been shown to influence fibroblast activation and ECM production [50].

Fibroblasts and myofibroblasts are mesenchymal cells directly involved in the deposition of ECM and collagen in the lung parenchyma. Fibroblasts may proliferate from inappropriate cytokine-driven activation or cell senescence [23]. The role of extracellular vesicles (EVs) in mediating fibroblast dysfunction is an emerging area of research [51,52]. EVs are lipid-bound vesicles secreted by cells into the extracellular space and are derived from various cell types, including macrophages and epithelial cells [53]. Fibroblasts exposed to EVs containing fibronectin exhibit increased migratory capacity and invasive behavior [54]. Increased stiffness of the lung may also promote fibroblast activation and myofibroblast differentiation through mechanotransduction pathways [55,56].

### 2.3. Cellular Mediators of Fibrosis

Cyclic adenosine monophosphate (cAMP) is a hydrophilic intracellular secondary messenger molecule involved in the regulation of multiple cellular processes [57]. Loss or dysregulation of cAMP signaling has been associated with the development or progression of fibrosis through fibroblast activation, inflammation, and ECM remodeling [58]. cAMP primarily exerts its effects via the activation of protein kinase A and other intracellular pathways which better regulate fibroblast function and reduce ECM deposition [59]. Attenuation of fibrosis has also been linked to decreased TGF-β signaling, where increased cAMP signaling has been shown to inhibit TGF-β production and activation [60]. Elevated cAMP levels have also been shown to inhibit the production of pro-inflammatory cytokines and chemokines, reducing inflammation in the lung and potentially attenuating pro-fibrotic pathways [61,62]. Phosphodiesterase 4 (PDE4) inhibitors, which increase intracellular cAMP levels, have been shown to decrease fibroblast proliferation and myofibroblast differentiation [63].

Lysophosphatidic acid (LPA) is a bioactive lipid mediator involved in various cellular processes including cell proliferation, migration, and survival [64]. It is synthesized by autotaxin, an enzyme that catalyzes the hydrolysis of lysophosphatidylcholine to LPA, the latter serving as the primary ligand for lysophosphatidic acid receptor 1 (LPAR1) [65]. The activation of LPAR1 is associated with several intracellular signaling pathways in fibrosis including those promoting cytoskeletal rearrangements, and fibroblast migration and proliferation [66,67]. LPAR1 also activates TGF-β and other pro-fibrotic mediators [68], with LPAR1 signaling involved in the recruitment and activation of inflammatory macrophages [69].

Integrins are transmembrane glycoprotein receptors that serve as mediators of cell adhesion, communication, and membrane integrity. Interplay between integrins and TGF-β has been associated with aberrant induction of pro-fibrotic pathways [70]. Integrin αVβ6, found predominantly in epithelial cells, induces TGF-β activation after cell injury [71], while integrin α3β1 has been associated with EMT and myofibroblast proliferation in murine models of bleomycin-induce fibrosis [72].

### 2.4. Dysregulation of Extra-Cellular Matrix

Dysregulation of ECM homeostasis with an imbalance between synthesis and degradation of ECM components may result in inappropriate accumulation of fibrotic tissue [73,74]. Collagen (types I and III) is an essential protein in the ECM of lung tissue and is deposited by activated fibroblasts and myofibroblasts derived from resident or circulating fibrocytes, and epithelial cells undergoing EMT [75,76]. Excessive accumulation of collagen leads to the thickening of alveolar walls and the formation of fibroblast foci. Other ECM proteins and cytokines, including fibronectin and connective tissue growth factor (CTGF), have been associated with the formation of fibrotic tissue. Fibronectin is an adhesive glycoprotein crucial for cell adhesion, migration, and ECM organization, while CTGF is induced by TGF-β [77] and further promotes fibroblast proliferation and ECM production. ECM may also contribute to fibroblast proliferation through mechanotransduction pathways [78] and increased cell senescence [79].

The role of matrix metalloproteinases (MMPs) in ECM remodeling has been associated with disease progression in IPF. MMPs are enzymes responsible for the breakdown of ECM components, with their dysregulation leading to imbalances between ECM synthesis and degradation [80,81]. Elevated levels of MMPs in serum and bronchoalveolar lavage fluid (BAL) have been correlated with disease severity and progression [82,83,84]. In one study, BAL MMP-7 (matrilysin) levels were similarly elevated in IPF and non-IPF interstitial lung diseases with IPF distinguished by MMP-7 immunoreactivity in areas of parenchymal fibrosis compared to areas of acute inflammation in non-IPF diseases [84]. TGF-β is a key regulator of MMP expression in mice models of lung fibrosis, in particular limiting the activity of MMP-2 and MMP-9, which contributes to the aberrant accumulation of ECM proteins [85,86]. MMP-9 has also been shown to induce TGF-β production in human airway epithelium in one in vivo study [87], while MMP-12 acts as a downstream mediator of TGF-β-induced inflammation and fibrosis [88]. Its expression appears upregulated in response to TGF-β [89], though subsequent MMP-12 negative mice models did not demonstrate attenuated fibrotic responses to bleomycin lung injury [90]. Hyaluronan (HA) is a major component of the ECM in most organs. It is a glycosaminoglycan previously believed to be inert, but may play a regulatory role in the ECM of lung submucosa and alveoli [91]. HA is broken down by lysosomal enzymes known as hyaluronidases with prior studies demonstrating elevated levels of HA in the BAL of patients with IPF compared to controls [92,93]. Higher BAL levels of HA were also associated with worse clinical disease or progression [92]. Prior studies suggest HA-directed pathways may promote fibroblast proliferation and invasion [94,95], with HA synthases also driving fibroblast senescence [96].

### 2.5. Oxidative Stress, Mitochondria, and Macrophages

Imbalances between reactive oxygen species (ROS) production and antioxidants resulting in excessive oxidative injury have been recognized as a significant contributor to pro-fibrotic pathways [27]. Oxidative injury commonly damages epithelial cells and enhances TGF-β signaling [97]. Environmental triggers such as cigarette smoke [40] or cyclic mechanical stretch as seen with mechanical ventilation [98] have been associated with oxidative injury. Mitochondrial dysfunction is increasingly recognized as a contributor to profibrotic pathways [99]. While involved in the regulation of apoptosis [43], oxidative stress, and cellular metabolism, mitochondria also produce ROS as byproducts of oxidative phosphorylation [100].

Recent studies have highlighted the role of macrophage polarization and pro-inflammatory responses in IPF. In this setting, macrophages are categorized as either M1 (pro-inflammatory) or M2 (anti-inflammatory or profibrotic) [101]. M1 macrophages are typically activated in response to pathogens and tissue damage, releasing pro-inflammatory cytokines that may further exacerbate lung injury. M2 macrophages are associated with tissue repair and remodeling but promote fibrosis through activating the secretion of growth factors and ECM components by AECs and fibroblasts [102]. An imbalance between these two phenotypes may play a role in cellular responses to lung injury with activated macrophages also secreting TGF-β [47]. Lastly, macrophages also produce ROS [103] and increase inflammatory responses associated with fibrosis through activation of AIM2 inflammasomes [104].

### 2.6. Cell Senescence

Cell senescence in IPF is characterized by irreversible cell cycle arrest and secretion of pro-inflammatory factors, collectively known as the senescence-associated secretory phenotype [105,106]. This phenomenon affects various cells in the lung, including AECs [107], fibroblasts [23], and macrophages [108], all with particular roles in the progression and severity of fibrosis. Prior studies have also highlighted the role of specific signaling pathways in mediating the effects of cellular senescence in lung fibrosis [107,109]. β-catenin/p53 pathways have been shown to induce senescence in A549 cells (an AEC model) in response to various stressors [110]. Senescent AECs also exhibit increased expression of p16INK4a and p21WAF1/CIP1 markers, which have been associated with cell cycle arrest [111]. TGF-β signaling is a significant contributor to AEC senescence [112,113], promoting their transition to myofibroblast-like phenotypes and enhancing their fibrotic response.

**Table 1:** Selected mechanisms of fibrosis in idiopathic pulmonary fibrosis.

Mechanism Type	Summary	Key References
Alveolar epithelial injury and dysregulation	Damage to the alveolar epithelium and type II alveolar epithelial cells (AECs) may lead to aberrant pro-fibrotic responses, including excessive release of TGF-β, epithelial-mesenchymal transition (EMT), and inappropriate senescence of AECs	[24,28,31,33,35,42]
TGF-β, Fibroblasts and Myofibroblast dysfunction	Aberrant and sustained release of TGF-β may promote fibroblast and myofibroblast activation resulting in excessive extra-cellular matrix (ECM) deposition	[23,30,45,56]
Cellular Mediators of Fibrosis	Intracellular and extracellular mechanisms of fibrosis involve multiple pathways, including intracellular messenger molecule cyclic adenosine monophosphate (cAMP), lipid mediator lysophosphatidic acid (LPA), and cellular integrins or transmembrane glycoproteins, serving key roles in aspects of TGF-β release, fibroblast activation, proliferation, or migration, and inflammation.	[58,59,61,65,67,70]
Dysregulation of Extra-cellular matrix	Abnormal ECM homeostasis is characterized by an imbalance between fibroblast and myofibroblast activity with aberrant ECM deposition and dysregulated matrix metalloproteinases, which are ineffective in the breaking down of ECM.	[74,79,81,85,94,96]
Oxidative Stress, Mitochondria, and Macrophages	Oxidative stress is a common trigger of AEC injury and may result from mitochondrial dysfunction, which is involved in the creation of reactive oxygen species through oxidative phosphorylation. Macrophage polarization is involved in promoting inflammatory and anti-inflammatory (or pro-fibrotic) pathways, leading to aberrant AEC and fibroblast responses to injury.	[97,99,101]
Cell Senescence	Inappropriate cell cycle arrest and its related pro-inflammatory phenotype may involve AECs, fibroblasts, and macrophages, resulting in persistent and inappropriate fibrotic responses to cell injury and repair.	[23,25,42,107,108,109]

## 3. Potential Medical Therapies for IPF

Novel approaches targeting mechanisms of fibrosis, as outlined above, form the basis of recent clinical studies in IPF. This section highlights selected agents being evaluated in ongoing or recently completed Phase I or higher comparative trials in the last few years (Table 2), with a focus on drug mechanism, safety profile, and potential efficacy.

### 3.1. Molecular Targets of Fibrotic Pathways

#### 3.1.1. BI 1015550 (Nerandomilast)

BI 1015550 is an oral phosphodiesterase 4EB (PDE4B) inhibitor that selectively targets the PDE4B subtype, a regulator of intracellular signaling pathways associated with inflammation and fibrosis [114]. Inhibition of PDE4B is associated with increased cAMP levels, a secondary messenger known to exert anti-inflammatory and antifibrotic effects [61].

Recent clinical trials have explored the efficacy and safety of BI 1015550 in patients with both IPF and non-IPF progressive fibrotic disease. FIBRONEER is a phase III, double-blind, randomized, placebo-controlled study, aiming to evaluate the effects of BI 1015550 over 52 weeks in those with or without concomitant antifibrotic therapies (NCT05321069). Results from a phase II trial indicate BI 1015550 may stabilize lung function as measured by forced vital capacity (FVC) at 12 weeks [115]. In addition to its antifibrotic effects, BI 1015550 may also exert immunomodulatory effects that attenuate fibrotic processes in the lung [116,117]. Studies have shown that PDE4 inhibitors reduce the production of pro-inflammatory cytokines, including TNF-α and interleukin-4 [118,119], which are implicated in the pathogenesis of IPF. The safety profile of BI 1015550 appears to suggest minimal or tolerable adverse effects. In phase I studies, the drug was relatively well tolerated among healthy subjects with adverse effects reported in 50% and 90% of those on selected dosing, dominated by diarrhea and headaches [120]. Adverse effects in the phase II study were reported in 65% and 73% of those on and not on background antifibrotics, respectively, again dominated by diarrhea or loose stools [115].

#### 3.1.2. Inhaled Treprostinil

Treprostinil is a stable analogue of prostacyclin, a potent vasodilator involved in the regulation of vascular tone and inhibition of platelet aggregation. Activation of prostacyclin receptors by treprostinil leads to increased intracellular cAMP, associated with the inhibition of fibroblast proliferation and ECM deposition [59]. It is administered specifically in this setting using a specialized device for inhalation, typically four times a day. This allows the drug to act directly on the lungs, which can have fewer systemic adverse effects.

Inhaled treprostinil has been shown to attenuate fibrotic responses in various preclinical models, including those induced by bleomycin [121,122]. Treprostinil’s efficacy in IPF is further supported by its ability to reconstitute mitochondrial organization and structure in cells affected by the disease [123]. Mitochondrial dysfunction is a recognized feature of IPF, leading to imbalances in oxidative damage and repair. Inhaled treprostinil is currently approved for secondary pulmonary hypertension associated with interstitial lung disease [124]. Post-hoc analysis of the INCREASE trial evaluating efficacy of inhaled treprostinil in patients with secondary pulmonary hypertension due to fibrotic interstitial lung disease, found lower incidence of FVC decline [125], six-minute walk distance decline, and cardiopulmonary hospitalizations [126]. Current clinical trials are exploring the efficacy and safety of inhaled treprostinil in patients with IPF targeting FVC endpoints. TETON [127] and TETON-PPF are phase 3 randomized controlled trials assessing the impact of inhaled treprostinil on FVC change at 52 weeks in IPF and progressive pulmonary fibrosis, respectively (NCT05255991 and NCT05943535). The adverse-effect profile of inhaled treprostinil appears to be tolerable, and manageable with supportive medications (cough, throat irritation, and headache). Adverse effects were noted in the majority of treated patients in the INCREASE study though similar in terms of frequency to placebo (93% vs. 91%) (predominantly cough (44%) and headache (28%)) [124].

#### 3.1.3. Anlotinib

Anlotinib is a multi-targeted tyrosine kinase inhibitor originally developed for the treatment of various cancers [128], but has also shown promising antifibrotic effects in preclinical models of both pulmonary and non-pulmonary fibrosis [129]. Anlotinib exerts its antifibrotic effects through the inhibition of several key signaling pathways leading to the attenuation of TGF-β. Anlotinib attenuates TGF-β1 signaling [129] and facilitates apoptosis of myofibroblasts with slowing of EMT pathways in AECs [130]. Anlotinib has also been shown to inhibit glycolysis in myofibroblasts decreasing their activation and proliferation [131]. A current multicenter placebo-controlled phase II to phase III study is ongoing assessing FVC endpoints and safety outcomes at 52 weeks (NCT05828953). Its safety profile is derived from its use as monotherapy in extended stage small-cell lung cancer, highlighted by hypertension, hand-foot syndrome, and fatigue [132].

#### 3.1.4. Saracatinib

Saracatinib, also known as AZD0530, is a dual inhibitor of tyrosine kinases c-Src and Bcr-Abl, originally developed for the treatment of cancer but granted orphan drug designation by the US Food and Drug Administration for testing in IPF [133]. Prior research has demonstrated saracatinib effectively blocks TGF-β-induced Src kinase activation [134] and reduces α-smooth muscle actin expression, a marker of myofibroblast activation [135]. In preclinical studies, saracatinib has shown promise in reducing lung fibrosis in various models, including those induced by bleomycin [136]. The safety profile of saracatinib has been evaluated in early-phase clinical trials for various cancers, suggesting overall tolerance [137]. A current phase I study is ongoing to assess safety and tolerance outcomes in patients with IPF at 24 weeks (NCT04598919).

#### 3.1.5. SHR-1906

SHR-1906 is a fully humanized monoclonal antibody targeting CTGF. CTGF is a key mediator of fibroblast proliferation and ECM deposition with elevated tissue expression and plasma levels seen in IPF correlating with disease severity and progression [77,138,139]. Notably, pamrevlumab, a recombinant monoclonal antibody against CTGF showed early promise in a phase II study with a well-tolerated adverse effect profile [140], but did not meet FVC study endpoints in a large phase III study [18]. SHR-1906 was assessed in healthy participants in a phase I study, demonstrating a favorable safety profile with manageable adverse effects [141]. A phase II study is underway to further assess safety and efficacy over 26 weeks (NCT05722964).

#### 3.1.6. BMS-986278

BMS-986278 is a selective antagonist of LPAR1 involved in the promotion of various fibrotic pathways. Its ligand, LPA, is associated with fibroblast activation, proliferation, and deposition of ECM. In addition to its fibrotic effects, LPA also promotes inflammatory pathways that may contribute to fibrosis. In animal models, treatment with BMS-986278 has been associated with decreased collagen accumulation and improved lung function [142,143]. The safety profile of BMS-986278 has been assessed in early-phase studies, noting minimal adverse effects and general tolerability [144]. A phase II study assessing percent predicted change in FVC over 26 weeks in both IPF and non-IPF progressive ILD was recently completed [145] (NCT06003426). Preliminary findings presented in abstract form suggested decreased rate of FVC decline with or without background antifibrotic use [146].This drug has received Breakthrough Therapy Designation from the US FDA for the treatment of progressive pulmonary fibrosis.

#### 3.1.7. HZN-825 (Fipaxalparant)

HZN-825 is a selective allosteric modulator of LPAR1, the primary receptor for LPA-mediated fibrotic pathways. Dysregulated LPAR1 signaling again has been implicated in the promotion of fibroblast activation and fibrotic responses to lung injury [67]. The safety profile of HZN-825 has been evaluated in healthy participants where it showed favorable tolerances [147]. Adverse events reported were generally mild to moderate. A prior clinical study in patients with diffuse cutaneous scleroderma assessing Rodnan skin thickness scores and safety as primary endpoints suggested general tolerance and improvement in skin scores, though not statistically significant [148]. A multicenter randomized placebo-controlled phase IIb study is ongoing assessing FVC change at 52 weeks in patients with IPF (NCT05032066).

#### 3.1.8. BBT-877

BBT-877 is a novel inhibitor of autotaxin, the primary enzyme involved in the synthesis of LPA. Prior research indicates BBT-877 effectively reduces LPA production in various models of fibrotic injury [149,150], particularly antifibrotic effects in bleomycin-induced fibrosis [151]. In vivo studies have demonstrated BBT-877 does not significantly impair cell viability, even at high concentrations, suggesting a favorable safety profile for potential clinical use [151]. Notably, a prior novel autotaxin inhibitor ziritaxeset showed promise in early phase studies for treatment of IPF but did not meet primary or secondary study endpoints in two large multicenter phase III studies [16]. A phase II study with BBT-877 is ongoing, targeting absolute change in FVC over 24 weeks in patients with IPF (NCT05483907)

### 3.2. Integrin Inhibition

#### Bexotegrast or PLN-74809

Bexotegrast is an oral dual inhibitor of integrins αvβ6 and αvβ3, involved in several fibrotic pathways in the lung. Known to activate TGF-β, they play a constitutional role in promoting fibroblast proliferation and differentiation into myofibroblasts [72,152]. Preclinical studies have demonstrated bexotegrast may effectively inhibit TGF-β activation and reduce collagen deposition in lung fibroblast cultures and animal models of lung fibrosis [153]. Notably, a prior study assessing the efficacy of a subcutaneous monoclonal antibody inhibitor of αvβ6 failed to meet study endpoints due to early termination [154]. A multicenter phase IIb clinical trial assessing absolute change in FVC over 52 weeks in patients with IPF is ongoing for bexotegrast (NCT06097260).

### 3.3. Anti-Inflammatory Pathways

#### 3.3.1. Axatilimab

Axatilimab, also known as Niktimvo, is a humanized IgG4 monoclonal antibody that targets colony stimulating factor-1 receptor (CSF-1R), a cell surface protein thought to control the survival and function of monocytes and macrophages [155]. Axatilimab is involved in modulation of macrophage activity, believed to be a contributor in various fibrotic diseases, including IPF. In pre-clinical models, inhibition of signaling through the CSF-1 receptor was shown to reduce the number of disease-mediating macrophages along with their monocyte precursors [156,157]. Axatilimab has been primarily studied in the context of chronic graft-versus-host disease, showing promise in preclinical models and controlled studies for ameliorating fibrotic responses associated with this immune-mediated process [158,159]. A phase II clinical study is ongoing with absolute decline in FVC over 26 weeks as a primary outcome (NCT06132256).

#### 3.3.2. Jaktinib

The JAK-STAT signaling pathway is associated with the regulation of various cytokines and growth factors contributing to fibrosis in IPF [160]. Inhibiting JAK activity may reduce signaling of these profibrotic cytokines, thereby attenuating fibroblast activation and collagen deposition in the lungs. Jaktinib is a Janus kinase (JAK) inhibitor with potential therapeutic applications in various inflammatory and fibrotic diseases [161,162]. Jaktinib may influence immune response in the lungs and attenuate the activity of immune cells which contribute to fibrosis [163,164]. The safety profile of Jaktinib has been evaluated in early-phase clinical trials in myelofibrosis and hepatic fibrosis, where it has demonstrated a favorable tolerance among participants [161,165]. Common adverse effects associated with JAK inhibitors include gastrointestinal disturbances, increased risk of infections, and hematologic abnormalities. A phase II study assessing its anti-inflammatory and antifibrotic effects in IPF is ongoing, targeting FVC% change over 26 weeks (NCT04312594).

### 3.4. Oxidative Pathways

#### GKT137831 (Setanaxib)

GKT137831 is a selective inhibitor of nicotinamide adenine dinucleotide phosphate (NADPH) oxidases 1 (NOX1) and 4 (NOX4), which reduces the production of ROS and oxidative injury resulting in the activation of fibroblasts by damaged type II AECs. Preclinical studies have demonstrated GKT137831 effectively inhibits NOX1 and NOX4, leading to reduced oxidative stress and subsequent fibrosis in various fibrosis models [166]. GKT137831 has been shown to attenuate fibrosis associated with primary biliary cholangitis as seen in a phase II clinical study [167]. Similar NOX inhibitors have been evaluated in animal models of lung fibrosis, showing reduction in collagen deposition and improved lung function [168]. The compound’s ability to modulate TGF-β signaling pathways may be a key mechanism for its antifibrotic effects [169,170]. A current phase II study in patients with IPF is ongoing targeting a surrogate serum marker of oxidative stress at 24 weeks, along with secondary outcomes of FVC change, six-minute walk, and safety profile (NCT03865927).

### 3.5. Immune-Mediated or Cell-Cycle Pathways

#### 3.5.1. Tazarotene (GRI-0621)

Tazarotene, a synthetic retinoid which binds to retinoic acid receptors (RAR) RARβ and RARγ, was first developed for the topical treatment of plaque psoriasis [171]. Retinoids play important roles in regulating gene expression related to cell differentiation, proliferation, and apoptosis [172,173]. Tazarotene has been shown to induce apoptosis in various cell types, including cancer cells, through pathways that involve caspase activation and generation of ROS [174]. Tazarotene may also normalize cellular differentiation and counteract aberrant processes that lead to myofibroblast formation [173,175].

GRI-0621 is an oral formulation of tazarotene being assessed for its inhibitory effect on natural killer T-cells (NKT). Recent research has highlighted the role of type I NKT or invariant NKT (iNKT) cell types in the propagation of fibrosis [176,177]. Aberrant NKT activity or its absence in pre-clinical models of fibrosis have suggested both pro- and inhibitory effects on fibrotic pathways [178]. In a murine bleomycin-induced model of lung fibrosis, manipulation of iNKT-released IFN-γ resulted in ameliorated TGF-β–dependent fibrosis [179]. iNKT also mediates Th2 pathways attenuating the polarization of macrophages towards pro-fibrotic M2 phenotypes [180]. A phase 2a randomized controlled study is ongoing, assessing the efficacy of GRI-0621 in iNKT targeting primary and secondary endpoints of safety and changes in serologic biomarkers of NKT activity at 12 weeks (NCT06331624).

#### 3.5.2. Atezolizumab

Atezolizumab is a monoclonal antibody that inhibits programmed death-ligand 1 (PD-L1), a surface glycoprotein found on immune cells involved in cell proliferation and apoptosis [181]. The PD-1/PD-L1 pathway is crucial in regulating immune responses, particularly in the context of T-cell activation and tolerance [182,183]. Elevated serum levels of PD-1 and PD-L2 have been found in patients with systemic sclerosis and are associated with severity of lung fibrosis [184]. Elevated PD-L1 activity was detected in explanted fibroblasts of patients with IPF [185] while alveolar epithelial cells with increased surface markers of abnormal check-point activity were identified compared to normal donors [186]. Humanized murine mesenchymal stem cells were impacted by PD-1 pathways in ameliorating lung fibrosis [187]. Preclinical studies have indicated that immune checkpoint inhibitors (ICI), including atezolizumab, may modulate fibrotic responses in various models of lung injury [188,189].

An important concern for the use of atezolizumab in patients with chronic lung disease is the known association of ICI with pulmonary toxicity. Grades 1–4 pulmonary toxicity occurred in 16% of all patients treated with ICI or ICI-containing regimens in one large systematic review, with 6% being grades 4 or higher [190]. Described risk factors include underlying lung disease, particularly baseline fibrosis, which has been associated with higher mortality while on ICI therapy [191]. Risk of pneumonitis in real world settings may also be increased with combination therapies and radiation [192]. Incidence of pneumonitis in patients with non-small cell lung carcinoma and underlying interstitial lung disease treated with atezolizumab as monotherapy was 29.4% in one phase II, with the majority grade 3 or higher in terms of severity [193]. An assessment of anti-inflammatory and antifibrotic effects from ICI will need to be weighed against its pulmonary and other organ toxicity, particularly as long-term use may be necessary for managing chronic fibrosing diseases. A current Phase I study is ongoing to assess safety and efficacy of atezolizumab in patients with IPF over 24 weeks (NCT05515627).

## 4. Conclusions

IPF is a complex disease highlighted by aberrations in alveolar epithelial response to injury, inappropriate cytokine and chemokine-driven proliferation of fibroblasts and myofibroblasts, dysregulation of epithelial and mesenchymal interplay, and abnormal cell senescence. Novel agents targeting these various mechanisms are ongoing with challenges remaining in translating pre-clinical or molecular studies to in vivo or real-world effectiveness. A review of current concepts regarding the pathogenesis of IPF suggests interacting and overlapping pathways with complex up and downstream effects, making the manipulation or control of one pathway or target often insufficient for clinical effect. Current studies are assessing the impact of combination therapy with background use of approved antifibrotics, though future studies are likely needed to additionally engage additive or synergistic effects of multiple drugs as they become available. Lastly, adverse effects are common and will also be additive in the setting of future combination approaches. A multi-faceted approach may have greater therapeutic impact but will need to be tempered with higher risk of treatment-related toxicity. IPF, as currently diagnosed, is likely a heterogeneous entity, an aspect that needs further exploration in the search for optimal therapy.

## Figures and Tables

**Table 2:** Selected study agents and their mechanisms in the treatment of IPF.

NCT Number	Phase	Drug/Intervention	Mechanism Class
NCT05032066	2	HZN-825	LAPR1 receptor modulator, LPA pathway for fibrosis
NCT05321069	3	BI 1015550 (nerandomilast)	PDE4 inhibitor, fibrotic pathway reduction
NCT04312594	2	Jaktinib	JAK inhibitor, blocks JAK-induced pro-fibrotic cytokines, also anti-inflammatory
NCT05483907	2	BBT-877	autotaxin inhibitor, LPA pathway for fibrosis
NCT04598919	1	saracatinib	tyrosine kinase inhibitor, blocks TGF-β-induced Src kinase activation
NCT05255991	3	inhaled treprostinil	prostacyclin analogue, cAMP inhibition which has antifibrotic properties
NCT05515627	1	atezolizumab	immune checkpoint inhibitor, PDL-1 inhibitor for cell cycle
NCT06097260	2	bexotegrast (PLN-74809)	integrin inhibitor, blocks TGF-beta pathway
NCT06003426	3	BMS-986278	LPA receptor antagonist
NCT06132256	2	axatilimab	targets colony stimulating factor-1 receptor, or CSF-1R, anti-macrophage and monocyte therapy
NCT05722964	2	SHR-1906	monoclonal antibody against CTGF
NCT03865927	2	GKT137831 (setanaxib)	NADPH oxidase inhibitor
NCT06331624	2	Tazarotene (GRI-0621)	retinoic acid receptors binder, promotes apoptosis or stopping cell proliferation, iNKT inhibitor
NCT05828953	2 to 3	Anlotinib	tyrosine kinase inhibitor of TGF-β pathway, antifibrotic properties

Abbreviations: cAMP = cyclic adenosine monophosphate, CTGF = connective tissue growth factor, iNKT = invariant natural killer T-cell, LPA = lysophosphatidic acid, LAPR1 = lysophosphatidic acid receptor 1, NADPH = nicotinamide adenine dinucleotide phosphate, PDE4 = phosphodiesterase 4, PDL-1 = programmed death-ligand 1, TGF-β = transforming growth factor beta.

## Abbreviations

AEC	alveolar epithelial cell
cAMP	cyclic
CSF-1R	colony stimulating factor-1 receptor
CTGF	connective tissue growth factor
ECM	extracellular matrix
EMT	epithelial-mesenchymal transition
EV	extracellular vehicles
FVC	forced vital capacity
HA	hyaluronan
HRCT	high resolution chest CT
ICI	immune check point inhibitor
IPF	idiopathic pulmonary fibrosis
JAK	Janus kinase
LPA	lysophosphatidic acid
LPAR1	lysophosphatidic acid receptor 1
MMP	matrix metalloproteinases
NADPH	nicotinamide adenine dinucleotide phosphate
NKT	natural killer T-cells
NOX	NADPH oxidase
PDE	phosphodiesterase
PD-L1	programmed death-ligand 1
PFT	pulmonary function test
RAR	retinoic acid receptors
ROS	reactive oxygen species
TGF-β	transforming growth factor-beta
TNF-α	tumor necrosis factor-alpha

## References

[1] Raghu G., Remy-Jardin M., Richeldi L., Thomson C.C., Inoue Y., Johkoh T., Kreuter M., Lynch D.A., Maher T.M., Martinez F.J. (2022). Idiopathic Pulmonary Fibrosis (an Update) and Progressive Pulmonary Fibrosis in Adults: An Official ATS/ERS/JRS/ALAT Clinical Practice Guideline. Am. J. Respir. Crit. Care Med..

[2] King T.E., Bradford W.Z., Castro-Bernardini S., Fagan E.A., Glaspole I., Glassberg M.K., Gorina E., Hopkins P.M., Kardatzke D., Lancaster L. (2014). A phase 3 trial of pirfenidone in patients with idiopathic pulmonary fibrosis. N. Engl. J. Med..

[3] Richeldi L., du Bois R.M., Raghu G., Azuma A., Brown K.K., Costabel U., Cottin V., Flaherty K.R., Hansell D.M., Inoue Y. (2014). Efficacy and safety of nintedanib in idiopathic pulmonary fibrosis. N. Engl. J. Med..

[4] Iommi M., Gonnelli F., Bonifazi M., Faragalli A., Mei F., Pompili M., Carle F., Gesuita R. (2024). Understanding Patterns of Adherence to Antifibrotic Treatment in Idiopathic Pulmonary Fibrosis: Insights from an Italian Prospective Cohort Study. J. Clin. Med..

[5] Ruaro B., Salotti A., Reccardini N., Kette S., Da Re B., Nicolosi S., Zuccon U., Confalonieri M., Mondini L., Pozzan R. (2024). Functional Progression after Dose Suspension or Discontinuation of Nintedanib in Idiopathic Pulmonary Fibrosis: A Real-Life Multicentre Study. Pharmaceuticals.

[6] Idiopathic Pulmonary Fibrosis Clinical Research N., Raghu G., Anstrom K.J., King T.E., Lasky J.A., Martinez F.J. (2012). Prednisone, azathioprine, and N-acetylcysteine for pulmonary fibrosis. N. Engl. J. Med..

[7] Raghu G., Brown K.K., Bradford W.Z., Starko K., Noble P.W., Schwartz D.A., King T.E., Idiopathic Pulmonary Fibrosis Study G. (2004). A placebo-controlled trial of interferon gamma-1b in patients with idiopathic pulmonary fibrosis. N. Engl. J. Med..

[8] Raghu G., Brown K.K., Costabel U., Cottin V., du Bois R.M., Lasky J.A., Thomeer M., Utz J.P., Khandker R.K., McDermott L. (2008). Treatment of idiopathic pulmonary fibrosis with etanercept: An exploratory, placebo-controlled trial. Am. J. Respir. Crit. Care Med..

[9] Raghu G., Million-Rousseau R., Morganti A., Perchenet L., Behr J., Group M.S. (2013). Macitentan for the treatment of idiopathic pulmonary fibrosis: The randomised controlled MUSIC trial. Eur. Respir. J..

[10] King T.E., Behr J., Brown K.K., du Bois R.M., Lancaster L., de Andrade J.A., Stahler G., Leconte I., Roux S., Raghu G. (2008). BUILD-1: A randomized placebo-controlled trial of bosentan in idiopathic pulmonary fibrosis. Am. J. Respir. Crit. Care Med..

[11] Raghu G., Behr J., Brown K.K., Egan J.J., Kawut S.M., Flaherty K.R., Martinez F.J., Nathan S.D., Wells A.U., Collard H.R. (2013). Treatment of idiopathic pulmonary fibrosis with ambrisentan: A parallel, randomized trial. Ann. Intern. Med..

[12] Idiopathic Pulmonary Fibrosis Clinical Research N., Zisman D.A., Schwarz M., Anstrom K.J., Collard H.R., Flaherty K.R., Hunninghake G.W. (2010). A controlled trial of sildenafil in advanced idiopathic pulmonary fibrosis. N. Engl. J. Med..

[13] Daniels C.E., Lasky J.A., Limper A.H., Mieras K., Gabor E., Schroeder D.R., Imatinib I.P.F.S.I. (2010). Imatinib treatment for idiopathic pulmonary fibrosis: Randomized placebo-controlled trial results. Am. J. Respir. Crit. Care Med..

[14] Demedts M., Behr J., Buhl R., Costabel U., Dekhuijzen R., Jansen H.M., MacNee W., Thomeer M., Wallaert B., Laurent F. (2005). High-dose acetylcysteine in idiopathic pulmonary fibrosis. N. Engl. J. Med..

[15] Noth I., Anstrom K.J., Calvert S.B., de Andrade J., Flaherty K.R., Glazer C., Kaner R.J., Olman M.A., Idiopathic Pulmonary Fibrosis Clinical Research N. (2012). A placebo-controlled randomized trial of warfarin in idiopathic pulmonary fibrosis. Am. J. Respir. Crit. Care Med..

[16] Maher T.M., Ford P., Brown K.K., Costabel U., Cottin V., Danoff S.K., Groenveld I., Helmer E., Jenkins R.G., Milner J. (2023). Ziritaxestat, a Novel Autotaxin Inhibitor, and Lung Function in Idiopathic Pulmonary Fibrosis: The ISABELA 1 and 2 Randomized Clinical Trials. JAMA.

[17] Raghu G., Brown K.K., Collard H.R., Cottin V., Gibson K.F., Kaner R.J., Lederer D.J., Martinez F.J., Noble P.W., Song J.W. (2017). Efficacy of simtuzumab versus placebo in patients with idiopathic pulmonary fibrosis: A randomised, double-blind, controlled, phase 2 trial. Lancet Respir. Med..

[18] Raghu G., Richeldi L., Fernandez Perez E.R., De Salvo M.C., Silva R.S., Song J.W., Ogura T., Xu Z.J., Belloli E.A., Zhang X. (2024). Pamrevlumab for Idiopathic Pulmonary Fibrosis: The ZEPHYRUS-1 Randomized Clinical Trial. JAMA.

[19] Phan T.H.G., Paliogiannis P., Nasrallah G.K., Giordo R., Eid A.H., Fois A.G., Zinellu A., Mangoni A.A., Pintus G. (2021). Emerging cellular and molecular determinants of idiopathic pulmonary fibrosis. Cell. Mol. Life Sci..

[20] Kim K.I., Hossain R., Li X., Lee H.J., Lee C.J. (2023). Searching for Novel Candidate Small Molecules for Ameliorating Idiopathic Pulmonary Fibrosis: A Narrative Review. Biomol. Ther..

[21] Estornut C., Milara J., Bayarri M.A., Belhadj N., Cortijo J. (2021). Targeting Oxidative Stress as a Therapeutic Approach for Idiopathic Pulmonary Fibrosis. Front. Pharmacol..

[22] Aversa Z., Atkinson E.J., Carmona E.M., White T.A., Heeren A.A., Jachim S.K., Zhang X., Cummings S.R., Chiarella S.E., Limper A.H. (2023). Biomarkers of cellular senescence in idiopathic pulmonary fibrosis. Respir. Res..

[23] Lin Y., Xu Z. (2020). Fibroblast Senescence in Idiopathic Pulmonary Fibrosis. Front. Cell Dev. Biol..

[24] Chambers R.C., Mercer P.F. (2015). Mechanisms of alveolar epithelial injury, repair, and fibrosis. Ann. Am. Thorac. Soc..

[25] Hansel C., Jendrossek V., Klein D. (2020). Cellular Senescence in the Lung: The Central Role of Senescent Epithelial Cells. Int. J. Mol. Sci..

[26] Kristensen J.H., Karsdal M.A., Genovese F., Johnson S., Svensson B., Jacobsen S., Hagglund P., Leeming D.J. (2014). The role of extracellular matrix quality in pulmonary fibrosis. Respiration.

[27] Cameli P., Carleo A., Bergantini L., Landi C., Prasse A., Bargagli E. (2020). Oxidant/Antioxidant Disequilibrium in Idiopathic Pulmonary Fibrosis Pathogenesis. Inflammation.

[28] Selman M., Pardo A. (2020). The leading role of epithelial cells in the pathogenesis of idiopathic pulmonary fibrosis. Cell Signal..

[29] Ptasinski V.A., Stegmayr J., Belvisi M.G., Wagner D.E., Murray L.A. (2021). Targeting Alveolar Repair in Idiopathic Pulmonary Fibrosis. Am. J. Respir. Cell Mol. Biol..

[30] Kwong K.Y., Literat A., Zhu N.L., Huang H.H., Li C., Jones C.A., Minoo P. (2004). Expression of transforming growth factor beta (TGF-beta1) in human epithelial alveolar cells: A pro-inflammatory mediator independent pathway. Life Sci..

[31] Xu Y.D., Hua J., Mui A., O’Connor R., Grotendorst G., Khalil N. (2003). Release of biologically active TGF-beta1 by alveolar epithelial cells results in pulmonary fibrosis. Am. J. Physiol. Lung Cell. Mol. Physiol..

[32] Sisson T.H., Mendez M., Choi K., Subbotina N., Courey A., Cunningham A., Dave A., Engelhardt J.F., Liu X., White E.S. (2010). Targeted injury of type II alveolar epithelial cells induces pulmonary fibrosis. Am. J. Respir. Crit. Care Med..

[33] Kalluri R., Weinberg R.A. (2009). The basics of epithelial-mesenchymal transition. J. Clin. Investig..

[34] Willis B.C., Liebler J.M., Luby-Phelps K., Nicholson A.G., Crandall E.D., du Bois R.M., Borok Z. (2005). Induction of epithelial-mesenchymal transition in alveolar epithelial cells by transforming growth factor-beta1: Potential role in idiopathic pulmonary fibrosis. Am. J. Pathol..

[35] Salton F., Volpe M.C., Confalonieri M. (2019). Epithelial(-)Mesenchymal Transition in the Pathogenesis of Idiopathic Pulmonary Fibrosis. Medicina.

[36] Hill C., Jones M.G., Davies D.E., Wang Y. (2019). Epithelial-mesenchymal transition contributes to pulmonary fibrosis via aberrant epithelial/fibroblastic cross-talk. J. Lung Health Dis..

[37] Hackett T.L., Warner S.M., Stefanowicz D., Shaheen F., Pechkovsky D.V., Murray L.A., Argentieri R., Kicic A., Stick S.M., Bai T.R. (2009). Induction of epithelial-mesenchymal transition in primary airway epithelial cells from patients with asthma by transforming growth factor-beta1. Am. J. Respir. Crit. Care Med..

[38] Omori K., Hattori N., Senoo T., Takayama Y., Masuda T., Nakashima T., Iwamoto H., Fujitaka K., Hamada H., Kohno N. (2016). Inhibition of Plasminogen Activator Inhibitor-1 Attenuates Transforming Growth Factor-beta-Dependent Epithelial Mesenchymal Transition and Differentiation of Fibroblasts to Myofibroblasts. PLoS ONE.

[39] Michael S., Montag M., Dott W. (2013). Pro-inflammatory effects and oxidative stress in lung macrophages and epithelial cells induced by ambient particulate matter. Environ. Pollut..

[40] Aoshiba K., Nagai A. (2003). Oxidative stress, cell death, and other damage to alveolar epithelial cells induced by cigarette smoke. Tob. Induc. Dis..

[41] Tsuji T., Aoshiba K., Nagai A. (2004). Cigarette smoke induces senescence in alveolar epithelial cells. Am. J. Respir. Cell Mol. Biol..

[42] Parimon T., Chen P., Stripp B.R., Liang J., Jiang D., Noble P.W., Parks W.C., Yao C. (2023). Senescence of alveolar epithelial progenitor cells: A critical driver of lung fibrosis. Am. J. Physiol. Cell Physiol..

[43] Kim S.J., Cheresh P., Jablonski R.P., Williams D.B., Kamp D.W. (2015). The Role of Mitochondrial DNA in Mediating Alveolar Epithelial Cell Apoptosis and Pulmonary Fibrosis. Int. J. Mol. Sci..

[44] Cheng P., Li S., Chen H. (2021). Macrophages in Lung Injury, Repair, and Fibrosis. Cells.

[45] Frangogiannis N. (2020). Transforming growth factor-beta in tissue fibrosis. J. Exp. Med..

[46] Khalil N., Bereznay O., Sporn M., Greenberg A.H. (1989). Macrophage production of transforming growth factor beta and fibroblast collagen synthesis in chronic pulmonary inflammation. J. Exp. Med..

[47] Assoian R.K., Fleurdelys B.E., Stevenson H.C., Miller P.J., Madtes D.K., Raines E.W., Ross R., Sporn M.B. (1987). Expression and secretion of type beta transforming growth factor by activated human macrophages. Proc. Natl. Acad. Sci. USA.

[48] Verrecchia F., Mauviel A. (2002). Transforming growth factor-beta signaling through the Smad pathway: Role in extracellular matrix gene expression and regulation. J. Investig. Dermatol..

[49] Han Y.Y., Shen P., Chang W.X. (2015). Involvement of epithelial-to-mesenchymal transition and associated transforming growth factor-beta/Smad signaling in paraquat-induced pulmonary fibrosis. Mol. Med. Rep..

[50] Xiao L., Du Y., Shen Y., He Y., Zhao H., Li Z. (2012). TGF-beta 1 induced fibroblast proliferation is mediated by the FGF-2/ERK pathway. Front. Biosci. (Landmark Ed.).

[51] Kadota T., Yoshioka Y., Fujita Y., Araya J., Minagawa S., Hara H., Miyamoto A., Suzuki S., Fujimori S., Kohno T. (2020). Extracellular Vesicles from Fibroblasts Induce Epithelial-Cell Senescence in Pulmonary Fibrosis. Am. J. Respir. Cell Mol. Biol..

[52] Brigstock D.R. (2021). Extracellular Vesicles in Organ Fibrosis: Mechanisms, Therapies, and Diagnostics. Cells.

[53] Fujita Y. (2022). Extracellular vesicles in idiopathic pulmonary fibrosis: Pathogenesis and therapeutics. Inflamm. Regen..

[54] Chanda D., Otoupalova E., Hough K.P., Locy M.L., Bernard K., Deshane J.S., Sanderson R.D., Mobley J.A., Thannickal V.J. (2019). Fibronectin on the Surface of Extracellular Vesicles Mediates Fibroblast Invasion. Am. J. Respir. Cell Mol. Biol..

[55] Huang X., Yang N., Fiore V.F., Barker T.H., Sun Y., Morris S.W., Ding Q., Thannickal V.J., Zhou Y. (2012). Matrix stiffness-induced myofibroblast differentiation is mediated by intrinsic mechanotransduction. Am. J. Respir. Cell Mol. Biol..

[56] Hinz B. (2012). Mechanical aspects of lung fibrosis: A spotlight on the myofibroblast. Proc. Am. Thorac. Soc..

[57] Serezani C.H., Ballinger M.N., Aronoff D.M., Peters-Golden M. (2008). Cyclic AMP: Master regulator of innate immune cell function. Am. J. Respir. Cell Mol. Biol..

[58] Della Latta V., Cabiati M., Rocchiccioli S., Del Ry S., Morales M.A. (2013). The role of the adenosinergic system in lung fibrosis. Pharmacol. Res..

[59] Cash E., Goodwin A.T., Tatler A.L. (2023). Adenosine receptor signalling as a driver of pulmonary fibrosis. Pharmacol. Ther..

[60] Schiller M., Verrecchia F., Mauviel A. (2003). Cyclic adenosine 3′,5′-monophosphate-elevating agents inhibit transforming growth factor-beta-induced SMAD3/4-dependent transcription via a protein kinase A-dependent mechanism. Oncogene.

[61] Selige J., Hatzelmann A., Dunkern T. (2011). The differential impact of PDE4 subtypes in human lung fibroblasts on cytokine-induced proliferation and myofibroblast conversion. J. Cell. Physiol..

[62] Koga K., Takaesu G., Yoshida R., Nakaya M., Kobayashi T., Kinjyo I., Yoshimura A. (2009). Cyclic adenosine monophosphate suppresses the transcription of proinflammatory cytokines via the phosphorylated c-Fos protein. Immunity.

[63] Kolb M., Crestani B., Maher T.M. (2023). Phosphodiesterase 4B inhibition: A potential novel strategy for treating pulmonary fibrosis. Eur. Respir. Rev..

[64] Lin M.E., Herr D.R., Chun J. (2010). Lysophosphatidic acid (LPA) receptors: Signaling properties and disease relevance. Prostaglandins Other Lipid Mediat..

[65] Nakamura Y., Shimizu Y. (2023). Cellular and Molecular Control of Lipid Metabolism in Idiopathic Pulmonary Fibrosis: Clinical Application of the Lysophosphatidic Acid Pathway. Cells.

[66] Sakai N., Chun J., Duffield J.S., Wada T., Luster A.D., Tager A.M. (2013). LPA1-induced cytoskeleton reorganization drives fibrosis through CTGF-dependent fibroblast proliferation. FASEB J..

[67] Tang N., Zhao Y., Feng R., Liu Y., Wang S., Wei W., Ding Q., An M.S., Wen J., Li L. (2014). Lysophosphatidic acid accelerates lung fibrosis by inducing differentiation of mesenchymal stem cells into myofibroblasts. J. Cell. Mol. Med..

[68] Xu M.Y., Porte J., Knox A.J., Weinreb P.H., Maher T.M., Violette S.M., McAnulty R.J., Sheppard D., Jenkins G. (2009). Lysophosphatidic acid induces alphavbeta6 integrin-mediated TGF-beta activation via the LPA2 receptor and the small G protein G alpha(q). Am. J. Pathol..

[69] Jiang S., Yang H., Li M. (2023). Emerging Roles of Lysophosphatidic Acid in Macrophages and Inflammatory Diseases. Int. J. Mol. Sci..

[70] Goodwin A., Jenkins G. (2009). Role of integrin-mediated TGFbeta activation in the pathogenesis of pulmonary fibrosis. Biochem. Soc. Trans..

[71] Madala S.K., Korfhagen T.R., Schmidt S., Davidson C., Edukulla R., Ikegami M., Violette S.M., Weinreb P.H., Sheppard D., Hardie W.D. (2014). Inhibition of the alphavbeta6 integrin leads to limited alteration of TGF-alpha-induced pulmonary fibrosis. Am. J. Physiol. Lung Cell. Mol. Physiol..

[72] Kim K.K., Wei Y., Szekeres C., Kugler M.C., Wolters P.J., Hill M.L., Frank J.A., Brumwell A.N., Wheeler S.E., Kreidberg J.A. (2009). Epithelial cell alpha3beta1 integrin links beta-catenin and Smad signaling to promote myofibroblast formation and pulmonary fibrosis. J. Clin. Investig..

[73] Sonbol H.S. (2018). Extracellular Matrix Remodeling in Human Disease. J. Microsc. Ultrastruct..

[74] Upagupta C., Shimbori C., Alsilmi R., Kolb M. (2018). Matrix abnormalities in pulmonary fibrosis. Eur. Respir. Rev..

[75] Jessen H., Hoyer N., Prior T.S., Frederiksen P., Karsdal M.A., Leeming D.J., Bendstrup E., Sand J.M.B., Shaker S.B. (2021). Turnover of type I and III collagen predicts progression of idiopathic pulmonary fibrosis. Respir. Res..

[76] Snijder J., Peraza J., Padilla M., Capaccione K., Salvatore M.M. (2019). Pulmonary fibrosis: A disease of alveolar collapse and collagen deposition. Expert. Rev. Respir. Med..

[77] Leask A., Holmes A., Black C.M., Abraham D.J. (2003). Connective tissue growth factor gene regulation. Requirements for its induction by transforming growth factor-beta 2 in fibroblasts. J. Biol. Chem..

[78] Deng Z., Fear M.W., Suk Choi Y., Wood F.M., Allahham A., Mutsaers S.E., Prele C.M. (2020). The extracellular matrix and mechanotransduction in pulmonary fibrosis. Int. J. Biochem. Cell Biol..

[79] Blokland K.E.C., Pouwels S.D., Schuliga M., Knight D.A., Burgess J.K. (2020). Regulation of cellular senescence by extracellular matrix during chronic fibrotic diseases. Clin. Sci..

[80] Mahalanobish S., Saha S., Dutta S., Sil P.C. (2020). Matrix metalloproteinase: An upcoming therapeutic approach for idiopathic pulmonary fibrosis. Pharmacol. Res..

[81] Pardo A., Cabrera S., Maldonado M., Selman M. (2016). Role of matrix metalloproteinases in the pathogenesis of idiopathic pulmonary fibrosis. Respir. Res..

[82] Sokai A., Handa T., Tanizawa K., Oga T., Uno K., Tsuruyama T., Kubo T., Ikezoe K., Nakatsuka Y., Tanimura K. (2015). Matrix metalloproteinase-10: A novel biomarker for idiopathic pulmonary fibrosis. Respir. Res..

[83] Fujishima S., Shiomi T., Yamashita S., Yogo Y., Nakano Y., Inoue T., Nakamura M., Tasaka S., Hasegawa N., Aikawa N. (2010). Production and activation of matrix metalloproteinase 7 (matrilysin 1) in the lungs of patients with idiopathic pulmonary fibrosis. Arch. Pathol. Lab. Med..

[84] Vuorinen K., Myllarniemi M., Lammi L., Piirila P., Rytila P., Salmenkivi K., Kinnula V.L. (2007). Elevated matrilysin levels in bronchoalveolar lavage fluid do not distinguish idiopathic pulmonary fibrosis from other interstitial lung diseases. APMIS.

[85] Inoue R., Yasuma T., Fridman D’Alessandro V., Toda M., Ito T., Tomaru A., D’Alessandro-Gabazza C.N., Tsuruga T., Okano T., Takeshita A. (2023). Amelioration of Pulmonary Fibrosis by Matrix Metalloproteinase-2 Overexpression. Int. J. Mol. Sci..

[86] Bormann T., Maus R., Stolper J., Tort Tarres M., Brandenberger C., Wedekind D., Jonigk D., Welte T., Gauldie J., Kolb M. (2022). Role of matrix metalloprotease-2 and MMP-9 in experimental lung fibrosis in mice. Respir. Res..

[87] Perng D.W., Chang K.T., Su K.C., Wu Y.C., Chen C.S., Hsu W.H., Tsai C.M., Lee Y.C. (2011). Matrix metalloprotease-9 induces transforming growth factor-beta(1) production in airway epithelium via activation of epidermal growth factor receptors. Life Sci..

[88] Lagente V., Le Quement C., Boichot E. (2009). Macrophage metalloelastase (MMP-12) as a target for inflammatory respiratory diseases. Expert. Opin. Ther. Targets.

[89] Kang H.R., Cho S.J., Lee C.G., Homer R.J., Elias J.A. (2007). Transforming growth factor (TGF)-beta1 stimulates pulmonary fibrosis and inflammation via a Bax-dependent, bid-activated pathway that involves matrix metalloproteinase-12. J. Biol. Chem..

[90] Manoury B., Nenan S., Guenon I., Boichot E., Planquois J.M., Bertrand C.P., Lagente V. (2006). Macrophage metalloelastase (MMP-12) deficiency does not alter bleomycin-induced pulmonary fibrosis in mice. J. Inflamm..

[91] Lauer M.E., Dweik R.A., Garantziotis S., Aronica M.A. (2015). The Rise and Fall of Hyaluronan in Respiratory Diseases. Int. J. Cell Biol..

[92] Bjermer L., Lundgren R., Hallgren R. (1989). Hyaluronan and type III procollagen peptide concentrations in bronchoalveolar lavage fluid in idiopathic pulmonary fibrosis. Thorax.

[93] Milman N., Kristensen M.S., Bentsen K. (1995). Hyaluronan and procollagen type III aminoterminal peptide in serum and bronchoalveolar lavage fluid from patients with pulmonary fibrosis. APMIS.

[94] Li Y., Jiang D., Liang J., Meltzer E.B., Gray A., Miura R., Wogensen L., Yamaguchi Y., Noble P.W. (2011). Severe lung fibrosis requires an invasive fibroblast phenotype regulated by hyaluronan and CD44. J. Exp. Med..

[95] Xia H., Herrera J., Smith K., Yang L., Gilbertsen A., Benyumov A., Racila E., Bitterman P.B., Henke C.A. (2021). Hyaluronan/CD44 axis regulates S100A4-mediated mesenchymal progenitor cell fibrogenicity in idiopathic pulmonary fibrosis. Am. J. Physiol. Lung Cell. Mol. Physiol..

[96] Li Y., Liang J., Yang T., Monterrosa Mena J., Huan C., Xie T., Kurkciyan A., Liu N., Jiang D., Noble P.W. (2016). Hyaluronan synthase 2 regulates fibroblast senescence in pulmonary fibrosis. Matrix Biol..

[97] Kliment C.R., Oury T.D. (2010). Oxidative stress, extracellular matrix targets, and idiopathic pulmonary fibrosis. Free Radic. Biol. Med..

[98] Chapman K.E., Sinclair S.E., Zhuang D., Hassid A., Desai L.P., Waters C.M. (2005). Cyclic mechanical strain increases reactive oxygen species production in pulmonary epithelial cells. Am. J. Physiol. Lung Cell. Mol. Physiol..

[99] Bueno M., Calyeca J., Rojas M., Mora A.L. (2020). Mitochondria dysfunction and metabolic reprogramming as drivers of idiopathic pulmonary fibrosis. Redox Biol..

[100] Liu X., Chen Z. (2017). The pathophysiological role of mitochondrial oxidative stress in lung diseases. J. Transl. Med..

[101] Ge Z., Chen Y., Ma L., Hu F., Xie L. (2024). Macrophage polarization and its impact on idiopathic pulmonary fibrosis. Front. Immunol..

[102] Kishore A., Petrek M. (2021). Roles of Macrophage Polarization and Macrophage-Derived miRNAs in Pulmonary Fibrosis. Front. Immunol..

[103] Canton M., Sanchez-Rodriguez R., Spera I., Venegas F.C., Favia M., Viola A., Castegna A. (2021). Reactive Oxygen Species in Macrophages: Sources and Targets. Front. Immunol..

[104] Cho S.J., Hong K.S., Jeong J.H., Lee M., Choi A.M.K., Stout-Delgado H.W., Moon J.S. (2019). DROSHA-Dependent AIM2 Inflammasome Activation Contributes to Lung Inflammation during Idiopathic Pulmonary Fibrosis. Cells.

[105] Lopes-Paciencia S., Saint-Germain E., Rowell M.C., Ruiz A.F., Kalegari P., Ferbeyre G. (2019). The senescence-associated secretory phenotype and its regulation. Cytokine.

[106] Mebratu Y.A., Soni S., Rosas L., Rojas M., Horowitz J.C., Nho R. (2023). The aged extracellular matrix and the profibrotic role of senescence-associated secretory phenotype. Am. J. Physiol. Cell Physiol..

[107] Tu M., Wei T., Jia Y., Wang Y., Wu J. (2023). Molecular mechanisms of alveolar epithelial cell senescence and idiopathic pulmonary fibrosis: A narrative review. J. Thorac. Dis..

[108] Campbell R.A., Docherty M.H., Ferenbach D.A., Mylonas K.J. (2021). The Role of Ageing and Parenchymal Senescence on Macrophage Function and Fibrosis. Front. Immunol..

[109] Okuda R., Aoshiba K., Matsushima H., Ogura T., Okudela K., Ohashi K. (2019). Cellular senescence and senescence-associated secretory phenotype: Comparison of idiopathic pulmonary fibrosis, connective tissue disease-associated interstitial lung disease, and chronic obstructive pulmonary disease. J. Thorac. Dis..

[110] Han D., Xu Y., Peng W.P., Feng F., Wang Z., Gu C., Zhou X. (2021). Citrus Alkaline Extracts Inhibit Senescence of A549 Cells to Alleviate Pulmonary Fibrosis via the beta-Catenin/P53 Pathway. Med. Sci. Monit..

[111] Yamada Z., Nishio J., Motomura K., Mizutani S., Yamada S., Mikami T., Nanki T. (2022). Senescence of alveolar epithelial cells impacts initiation and chronic phases of murine fibrosing interstitial lung disease. Front. Immunol..

[112] Rana T., Jiang C., Liu G., Miyata T., Antony V., Thannickal V.J., Liu R.M. (2020). PAI-1 Regulation of TGF-beta1-induced Alveolar Type II Cell Senescence, SASP Secretion, and SASP-mediated Activation of Alveolar Macrophages. Am. J. Respir. Cell Mol. Biol..

[113] Minagawa S., Araya J., Numata T., Nojiri S., Hara H., Yumino Y., Kawaishi M., Odaka M., Morikawa T., Nishimura S.L. (2011). Accelerated epithelial cell senescence in IPF and the inhibitory role of SIRT6 in TGF-beta-induced senescence of human bronchial epithelial cells. Am. J. Physiol. Lung Cell. Mol. Physiol..

[114] Sisson T.H., Christensen P.J., Muraki Y., Dils A.J., Chibucos L., Subbotina N., Tohyama K., Horowitz J.C., Matsuo T., Bailie M. (2018). Phosphodiesterase 4 inhibition reduces lung fibrosis following targeted type II alveolar epithelial cell injury. Physiol. Rep..

[115] Richeldi L., Azuma A., Cottin V., Hesslinger C., Stowasser S., Valenzuela C., Wijsenbeek M.S., Zoz D.F., Voss F., Maher T.M. (2022). Trial of a Preferential Phosphodiesterase 4B Inhibitor for Idiopathic Pulmonary Fibrosis. N. Engl. J. Med..

[116] Sakkas L.I., Mavropoulos A., Bogdanos D.P. (2017). Phosphodiesterase 4 Inhibitors in Immune-mediated Diseases: Mode of Action, Clinical Applications, Current and Future Perspectives. Curr. Med. Chem..

[117] Spina D. (2003). Phosphodiesterase-4 inhibitors in the treatment of inflammatory lung disease. Drugs.

[118] Crilly A., Robertson S.E., Reilly J.H., Gracie J.A., Lai W.Q., Leung B.P., Life P.F., McInnes I.B. (2011). Phosphodiesterase 4 (PDE4) regulation of proinflammatory cytokine and chemokine release from rheumatoid synovial membrane. Ann. Rheum. Dis..

[119] Griswold D.E., Webb E.F., Badger A.M., Gorycki P.D., Levandoski P.A., Barnette M.A., Grous M., Christensen S., Torphy T.J. (1998). SB 207499 (Ariflo), a second generation phosphodiesterase 4 inhibitor, reduces tumor necrosis factor alpha and interleukin-4 production in vivo. J. Pharmacol. Exp. Ther..

[120] Maher T.M., Schlecker C., Luedtke D., Bossert S., Zoz D.F., Schultz A. (2022). Phase I studies of BI 1015550, a preferential phosphodiesterase 4B inhibitor, in healthy males and patients with idiopathic pulmonary fibrosis. ERJ Open Res..

[121] Nikitopoulou I., Manitsopoulos N., Kotanidou A., Tian X., Petrovic A., Magkou C., Ninou I., Aidinis V., Schermuly R.T., Kosanovic D. (2019). Orotracheal treprostinil administration attenuates bleomycin-induced lung injury, vascular remodeling, and fibrosis in mice. Pulm. Circ..

[122] Corboz M.R., Zhang J., LaSala D., DiPetrillo K., Li Z., Malinin V., Brower J., Kuehl P.J., Barrett T.E., Perkins W.R. (2018). Therapeutic administration of inhaled INS1009, a treprostinil prodrug formulation, inhibits bleomycin-induced pulmonary fibrosis in rats. Pulm. Pharmacol. Ther..

[123] Fang L., Chen W.C., Jaksch P., Molino A., Saglia A., Roth M., Lambers C. (2023). Treprostinil Reconstitutes Mitochondrial Organisation and Structure in Idiopathic Pulmonary Fibrosis Cells. Int. J. Mol. Sci..

[124] Waxman A., Restrepo-Jaramillo R., Thenappan T., Ravichandran A., Engel P., Bajwa A., Allen R., Feldman J., Argula R., Smith P. (2021). Inhaled Treprostinil in Pulmonary Hypertension Due to Interstitial Lung Disease. N. Engl. J. Med..

[125] Nathan S.D., Waxman A., Rajagopal S., Case A., Johri S., DuBrock H., De La Zerda D.J., Sahay S., King C., Melendres-Groves L. (2021). Inhaled treprostinil and forced vital capacity in patients with interstitial lung disease and associated pulmonary hypertension: A post-hoc analysis of the INCREASE study. Lancet Respir. Med..

[126] Nathan S.D., Tapson V.F., Elwing J., Rischard F., Mehta J., Shapiro S., Shen E., Deng C., Smith P., Waxman A. (2022). Efficacy of Inhaled Treprostinil on Multiple Disease Progression Events in Patients with Pulmonary Hypertension due to Parenchymal Lung Disease in the INCREASE Trial. Am. J. Respir. Crit. Care Med..

[127] Nathan S.D., Behr J., Cottin V., Lancaster L., Smith P., Deng C.Q., Pearce N., Bell H., Peterson L., Flaherty K.R. (2022). Study design and rationale for the TETON phase 3, randomised, controlled clinical trials of inhaled treprostinil in the treatment of idiopathic pulmonary fibrosis. BMJ Open Respir. Res..

[128] Chen S., Gao D., Sun R., Bao J., Lu C., Zhang Z., Xiao T., Gu X., Zhou H. (2023). Anlotinib prove to be a potential therapy for the treatment of pulmonary fibrosis complicated with lung adenocarcinoma. Pulm. Pharmacol. Ther..

[129] Wu Y.T., Li Q.Z., Zhao X.K., Mu M., Zou G.L., Zhao W.F. (2023). Anlotinib Attenuates Liver Fibrosis by Regulating the Transforming Growth Factor beta1/Smad3 Signaling Pathway. Dig. Dis. Sci..

[130] Ruan H., Lv Z., Liu S., Zhang L., Huang K., Gao S., Gan W., Liu X., Zhang S., Helian K. (2020). Anlotinib attenuated bleomycin-induced pulmonary fibrosis via the TGF-beta1 signalling pathway. J. Pharm. Pharmacol..

[131] Chen W., Zhang J., Zhong W., Liu Y., Lu Y., Zeng Z., Huang H., Wan X., Meng X., Zou F. (2021). Anlotinib Inhibits PFKFB3-Driven Glycolysis in Myofibroblasts to Reverse Pulmonary Fibrosis. Front. Pharmacol..

[132] Li Y., Sun Z., Sun W., Wang H., Zu J. (2022). Effectiveness and Safety of Anlotinib Monotherapy for Patients with Extensive-stage Small-Cell Lung Cancer Who Progressed to Chemotherapy: A Real-world Exploratory Study. Clin. Med. Insights Oncol..

[133] Ramos R., Vale N. (2024). Dual Drug Repurposing: The Example of Saracatinib. Int. J. Mol. Sci..

[134] Du G., Wang J., Zhang T., Ding Q., Jia X., Zhao X., Dong J., Yang X., Lu S., Zhang C. (2020). Targeting Src family kinase member Fyn by Saracatinib attenuated liver fibrosis in vitro and in vivo. Cell Death Dis..

[135] Hu M., Che P., Han X., Cai G.Q., Liu G., Antony V., Luckhardt T., Siegal G.P., Zhou Y., Liu R.M. (2014). Therapeutic targeting of SRC kinase in myofibroblast differentiation and pulmonary fibrosis. J. Pharmacol. Exp. Ther..

[136] Ahangari F., Becker C., Foster D.G., Chioccioli M., Nelson M., Beke K., Wang X., Justet A., Adams T., Readhead B. (2022). Saracatinib, a Selective Src Kinase Inhibitor, Blocks Fibrotic Responses in Preclinical Models of Pulmonary Fibrosis. Am. J. Respir. Crit. Care Med..

[137] Baselga J., Cervantes A., Martinelli E., Chirivella I., Hoekman K., Hurwitz H.I., Jodrell D.I., Hamberg P., Casado E., Elvin P. (2010). Phase I safety, pharmacokinetics, and inhibition of SRC activity study of saracatinib in patients with solid tumors. Clin. Cancer Res..

[138] Yanagihara T., Tsubouchi K., Gholiof M., Chong S.G., Lipson K.E., Zhou Q., Scallan C., Upagupta C., Tikkanen J., Keshavjee S. (2022). Connective-Tissue Growth Factor Contributes to TGF-beta1-induced Lung Fibrosis. Am. J. Respir. Cell Mol. Biol..

[139] Kono M., Nakamura Y., Suda T., Kato M., Kaida Y., Hashimoto D., Inui N., Hamada E., Miyazaki O., Kurashita S. (2011). Plasma CCN2 (connective tissue growth factor; CTGF) is a potential biomarker in idiopathic pulmonary fibrosis (IPF). Clin. Chim. Acta.

[140] Richeldi L., Fernandez Perez E.R., Costabel U., Albera C., Lederer D.J., Flaherty K.R., Ettinger N., Perez R., Scholand M.B., Goldin J. (2020). Pamrevlumab, an anti-connective tissue growth factor therapy, for idiopathic pulmonary fibrosis (PRAISE): A phase 2, randomised, double-blind, placebo-controlled trial. Lancet Respir. Med..

[141] Song L.L., Zhou H.Y., Ye P.P., Li Q., Chen K.G., Zhang Y.H., Zhao F.R., Shi J.Y., Luo Y., Zhu M. (2023). A first-in-human phase I study of SHR-1906, a humanized monoclonal antibody against connective tissue growth factor, in healthy participants. Clin. Transl. Sci..

[142] Murphy B., Sum C.-S., Wang T., Heiry R., Kalinowski S., Hung C.-P., Chu C.-H., Azzara A., Milinda Z., Burns L. (2019). LPA1 antagonist BMS-986278 for idiopathic pulmonary fibrosis: Preclinical pharmacological in vitro and in vivo evaluation. Eur. Respir. J..

[143] Cheng P.T., Kaltenbach III R.F., Zhang H., Shi J., Tao S., Li J., Kennedy L.J., Walker S.J., Shi Y., Wang Y. (2021). Discovery of an oxycyclohexyl acid lysophosphatidic acid receptor 1 (LPA1) antagonist BMS-986278 for the treatment of pulmonary fibrotic diseases. J. Med. Chem..

[144] Gill M.W., Murphy B.J., Cheng P.T., Sivaraman L., Davis M., Lehman-McKeeman L. (2022). Mechanism of hepatobiliary toxicity of the LPA1 antagonist BMS-986020 developed to treat idiopathic pulmonary fibrosis: Contrasts with BMS-986234 and BMS-986278. Toxicol. Appl. Pharmacol..

[145] Corte T.J., Lancaster L., Swigris J.J., Maher T.M., Goldin J.G., Palmer S.M., Suda T., Ogura T., Minnich A., Zhan X. (2021). Phase 2 trial design of BMS-986278, a lysophosphatidic acid receptor 1 (LPA(1)) antagonist, in patients with idiopathic pulmonary fibrosis (IPF) or progressive fibrotic interstitial lung disease (PF-ILD). BMJ Open Respir. Res..

[146] Corte T., Cottin V., Glassberg M., Kreuter M., Ogura T., Suda T., Goldin J., Berkowitz E., Elpers B., Kim S. (2023). BMS-986278, an oral lysophosphatidic acid receptor 1 (LPA1) antagonist, for patients with idiopathic pulmonary fibrosis: Results from a phase 2 randomized trial. B17. Emerging Data on Disease and Symptom Based Therapeutics for Patients with IPF.

[147] Song Y., Ali F.N., Ye Z., Zarzoso J., Rogowski J., Sun Y., Xin Y. (2024). Pharmacokinetics of Fipaxalparant, a Small-Molecule Selective Negative Allosteric Modulator of Lysophosphatidic Acid Receptor 1, and the Effect of Food in Healthy Volunteers. Clin. Pharmacol. Drug Dev..

[148] Allanore Y., Distler O., Jagerschmidt A., Illiano S., Ledein L., Boitier E., Agueusop I., Denton C.P., Khanna D. (2018). Lysophosphatidic Acid Receptor 1 Antagonist SAR100842 for Patients With Diffuse Cutaneous Systemic Sclerosis: A Double-Blind, Randomized, Eight-Week Placebo-Controlled Study Followed by a Sixteen-Week Open-Label Extension Study. Arthritis Rheumatol..

[149] Kim J.S., Shin M.J., Lee S.Y., Choi S.M., Choi K.U., Suh D.S., Kim D.K., Kim J.H. (2024). BBT-877, a Novel Autotaxin Inhibitor, Abrogates Drug Resistance in Epithelial Ovarian Cancer Stem Cells. Anticancer. Res..

[150] Lee J.H., Khin P.P., Lee G., Lim O.K., Jun H.S. (2022). Effect of BBT-877, a novel inhibitor of ATX, on a mouse model of type 1 diabetic nephropathy. Aging.

[151] Lee G., Kang S.U., Ryou J.-H., Lim J.-J., Lee D.-Y., Kwon H.-J., Ha G.-H., Lee Y.-H. (2019). BBT-877, a potent autotaxin inhibitor in clinical development to treat idiopathic pulmonary fibrosis. PAT.

[152] Bellani S., Molyneaux P.L., Maher T.M., Spagnolo P. (2024). Potential of alphavbeta6 and alphavbeta1 integrin inhibition for treatment of idiopathic pulmonary fibrosis. Expert. Opin. Ther. Targets.

[153] An M., Ho S., Rao V., Ho B., Ahn R., Yuzon J., Budi E., Lau J., Her C., Wolters P. (2024). Bexotegrast Targets TGF-β Inhibition to Specific Cell Types in the Fibrotic Human Lung. Artery.

[154] Raghu G., Mouded M., Chambers D.C., Martinez F.J., Richeldi L., Lancaster L.H., Hamblin M.J., Gibson K.F., Rosas I.O., Prasse A. (2022). A Phase IIb Randomized Clinical Study of an Anti-alpha(v)beta(6) Monoclonal Antibody in Idiopathic Pulmonary Fibrosis. Am. J. Respir. Crit. Care Med..

[155] Peyraud F., Cousin S., Italiano A. (2017). CSF-1R Inhibitor Development: Current Clinical Status. Curr. Oncol. Rep..

[156] Meziani L., Mondini M., Petit B., Boissonnas A., Thomas de Montpreville V., Mercier O., Vozenin M.C., Deutsch E. (2018). CSF1R inhibition prevents radiation pulmonary fibrosis by depletion of interstitial macrophages. Eur. Respir. J..

[157] Watson C.K., Schloesser D., Fundel-Clemens K., Lerner C., Gabler S., Baskaran P., Wohnhaas C.T., Dichtl S., Huber H.J., Ask K. (2023). Antifibrotic Drug Nintedanib Inhibits CSF1R to Promote IL-4-associated Tissue Repair Macrophages. Am. J. Respir. Cell Mol. Biol..

[158] Kitko C.L., Arora M., DeFilipp Z., Zaid M.A., Di Stasi A., Radojcic V., Betts C.B., Coussens L.M., Meyers M.L., Qamoos H. (2023). Axatilimab for Chronic Graft-Versus-Host Disease After Failure of at Least Two Prior Systemic Therapies: Results of a Phase I/II Study. J. Clin. Oncol..

[159] Wolff D., Cutler C., Lee S.J., Pusic I., Bittencourt H., White J., Hamadani M., Arai S., Salhotra A., Perez-Simon J.A. (2024). Axatilimab in Recurrent or Refractory Chronic Graft-versus-Host Disease. N. Engl. J. Med..

[160] Milara J., Hernandez G., Ballester B., Morell A., Roger I., Montero P., Escriva J., Lloris J.M., Molina-Molina M., Morcillo E. (2018). The JAK2 pathway is activated in idiopathic pulmonary fibrosis. Respir. Res..

[161] Zhang Y., Zhang Q., Liu Q., Dang H., Gao S., Wang W., Zhou H., Chen Y., Ma L., Wang J. (2023). Safety and efficacy of jaktinib (a novel JAK inhibitor) in patients with myelofibrosis who are relapsed or refractory to ruxolitinib: A single-arm, open-label, phase 2, multicenter study. Am. J. Hematol..

[162] Park E., Park S., Lee S.J., Jeong D., Jin H., Moon H., Cha B., Kim D., Ma S., Seo W. (2023). Identification and Biological Evaluation of a Potent and Selective JAK1 Inhibitor for the Treatment of Pulmonary Fibrosis. J. Med. Chem..

[163] Silva-Carmona M., Vogel T.P., Marchal S., Guesmi M., Dubus J.C., Leroy S., Fabre A., Barlogis V., Forbes L.R., Giovannini-Chami L. (2020). Successful Treatment of Interstitial Lung Disease in STAT3 Gain-of-Function Using JAK Inhibitors. Am. J. Respir. Crit. Care Med..

[164] Montero P., Milara J., Roger I., Cortijo J. (2021). Role of JAK/STAT in Interstitial Lung Diseases; Molecular and Cellular Mechanisms. Int. J. Mol. Sci..

[165] Zhao M., Zhang H., Ma S., Gong S., Wei C., Miao L., Zhao W. (2024). Clinical pharmacokinetic characteristics of Jaktinib in subjects with hepatic impairment in a phase I trial. Drug Metab. Pharmacokinet..

[166] Thannickal V.J., Jandeleit-Dahm K., Szyndralewiez C., Torok N.J. (2023). Pre-clinical evidence of a dual NADPH oxidase 1/4 inhibitor (setanaxib) in liver, kidney and lung fibrosis. J. Cell. Mol. Med..

[167] Invernizzi P., Carbone M., Jones D., Levy C., Little N., Wiesel P., Nevens F., the study investigators (2023). Setanaxib, a first-in-class selective NADPH oxidase 1/4 inhibitor for primary biliary cholangitis: A randomized, placebo-controlled, phase 2 trial. Liver Int..

[168] Hecker L., Vittal R., Jones T., Jagirdar R., Luckhardt T.R., Horowitz J.C., Pennathur S., Martinez F.J., Thannickal V.J. (2009). NADPH oxidase-4 mediates myofibroblast activation and fibrogenic responses to lung injury. Nat. Med..

[169] Louzada R.A., Corre R., Ameziane El Hassani R., Meziani L., Jaillet M., Cazes A., Crestani B., Deutsch E., Dupuy C. (2021). NADPH oxidase DUOX1 sustains TGF-beta1 signalling and promotes lung fibrosis. Eur. Respir. J..

[170] Jiang F., Liu G.S., Dusting G.J., Chan E.C. (2014). NADPH oxidase-dependent redox signaling in TGF-beta-mediated fibrotic responses. Redox Biol..

[171] Duvic M. (1997). Tazarotene: A review of its pharmacological profile and potential for clinical use in psoriasis. Expert. Opin. Investig. Drugs.

[172] Zhou T.B., Drummen G.P., Qin Y.H. (2012). The controversial role of retinoic acid in fibrotic diseases: Analysis of involved signaling pathways. Int. J. Mol. Sci..

[173] Tabata C., Kadokawa Y., Tabata R., Takahashi M., Okoshi K., Sakai Y., Mishima M., Kubo H. (2006). All-trans-retinoic acid prevents radiation- or bleomycin-induced pulmonary fibrosis. Am. J. Respir. Crit. Care Med..

[174] So P.L., Fujimoto M.A., Epstein E.H. (2008). Pharmacologic retinoid signaling and physiologic retinoic acid receptor signaling inhibit basal cell carcinoma tumorigenesis. Mol. Cancer Ther..

[175] Al Haj Zen A., Nawrot D.A., Howarth A., Caporali A., Ebner D., Vernet A., Schneider J.E., Bhattacharya S. (2016). The Retinoid Agonist Tazarotene Promotes Angiogenesis and Wound Healing. Mol. Ther..

[176] Calamita E., Liu W.H., Ogger P.P., Griffin L., Michalaki C., Murphy F., Worrell J., McCarthy C., Agro A., Hertz M. (2024). Type 1 Invariant Natural Killer T Cells Drive Lung Fibrosis. Am. J. Respir. Crit. Care Med..

[177] Kumar V., Hertz M., Agro A., Byrne A.J. (2023). Type 1 invariant natural killer T cells in chronic inflammation and tissue fibrosis. Front. Immunol..

[178] Jandl K., Marsh L.M., Mutgan A.C., Crnkovic S., Valzano F., Zabini D., Hoffmann J., Foris V., Gschwandtner E., Klepetko W. (2022). Impairment of the NKT-STAT1-CXCL9 Axis Contributes to Vessel Fibrosis in Pulmonary Hypertension Caused by Lung Fibrosis. Am. J. Respir. Crit. Care Med..

[179] Kim J.H., Kim H.Y., Kim S., Chung J.H., Park W.S., Chung D.H. (2005). Natural killer T (NKT) cells attenuate bleomycin-induced pulmonary fibrosis by producing interferon-gamma. Am. J. Pathol..

[180] Grabarz F., Aguiar C.F., Correa-Costa M., Braga T.T., Hyane M.I., Andrade-Oliveira V., Landgraf M.A., Camara N.O.S. (2018). Protective role of NKT cells and macrophage M2-driven phenotype in bleomycin-induced pulmonary fibrosis. Inflammopharmacology.

[181] Zhang F., Qi X., Wang X., Wei D., Wu J., Feng L., Cai H., Wang Y., Zeng N., Xu T. (2017). Structural basis of the therapeutic anti-PD-L1 antibody atezolizumab. Oncotarget.

[182] Jiang A., Liu N., Wang J., Zheng X., Ren M., Zhang W., Yao Y. (2022). The role of PD-1/PD-L1 axis in idiopathic pulmonary fibrosis: Friend or foe?. Front. Immunol..

[183] Kronborg-White S., Madsen L.B., Bendstrup E., Poletti V. (2021). PD-L1 Expression in Patients with Idiopathic Pulmonary Fibrosis. J. Clin. Med..

[184] Fukasawa T., Yoshizaki A., Ebata S., Nakamura K., Saigusa R., Miura S., Yamashita T., Hirabayashi M., Ichimura Y., Taniguchi T. (2017). Contribution of Soluble Forms of Programmed Death 1 and Programmed Death Ligand 2 to Disease Severity and Progression in Systemic Sclerosis. Arthritis Rheumatol..

[185] Geng Y., Liu X., Liang J., Habiel D.M., Kulur V., Coelho A.L., Deng N., Xie T., Wang Y., Liu N. (2019). PD-L1 on invasive fibroblasts drives fibrosis in a humanized model of idiopathic pulmonary fibrosis. JCI Insight.

[186] Ahmadvand N., Carraro G., Jones M.R., Shalashova I., Noori A., Wilhelm J., Baal N., Khosravi F., Chen C., Zhang J.S. (2022). Cell-Surface Programmed Death Ligand-1 Expression Identifies a Sub-Population of Distal Epithelial Cells Enriched in Idiopathic Pulmonary Fibrosis. Cells.

[187] Ni K., Liu M., Zheng J., Wen L., Chen Q., Xiang Z., Lam K.T., Liu Y., Chan G.C., Lau Y.L. (2018). PD-1/PD-L1 Pathway Mediates the Alleviation of Pulmonary Fibrosis by Human Mesenchymal Stem Cells in Humanized Mice. Am. J. Respir. Cell Mol. Biol..

[188] Zhao Y., Hao C., Li M., Qu Y., Guo Y., Deng X., Si H., Yao W. (2022). PD-1/PD-L1 inhibitor ameliorates silica-induced pulmonary fibrosis by maintaining systemic immune homeostasis. Biomed. Pharmacother..

[189] Lu Y., Zhong W., Liu Y., Chen W., Zhang J., Zeng Z., Huang H., Qiao Y., Wan X., Meng X. (2022). Anti-PD-L1 antibody alleviates pulmonary fibrosis by inducing autophagy via inhibition of the PI3K/Akt/mTOR pathway. Int. Immunopharmacol..

[190] Zhou P., Zhao X., Wang G. (2022). Risk Factors for Immune Checkpoint Inhibitor-Related Pneumonitis in Cancer Patients: A Systemic Review and Meta-Analysis. Respiration.

[191] Atchley W.T., Alvarez C., Saxena-Beem S., Schwartz T.A., Ishizawar R.C., Patel K.P., Rivera M.P. (2021). Immune Checkpoint Inhibitor-Related Pneumonitis in Lung Cancer: Real-World Incidence, Risk Factors, and Management Practices Across Six Health Care Centers in North Carolina. Chest.

[192] Su Q., Zhu E.C., Wu J.B., Li T., Hou Y.L., Wang D.Y., Gao Z.H. (2019). Risk of Pneumonitis and Pneumonia Associated With Immune Checkpoint Inhibitors for Solid Tumors: A Systematic Review and Meta-Analysis. Front. Immunol..

[193] Ikeda S., Kato T., Kenmotsu H., Ogura T., Iwasawa S., Sato Y., Harada T., Kubota K., Tokito T., Okamoto I. (2020). A Phase 2 Study of Atezolizumab for Pretreated NSCLC With Idiopathic Interstitial Pneumonitis. J. Thorac. Oncol..

